# Taking a Leap of Faith: A Study of Abruptly Transitioning an Undergraduate Medical Education Program to Distance-Learning Owing to the COVID-19 Pandemic

**DOI:** 10.2196/27010

**Published:** 2021-07-23

**Authors:** Stefan S Du Plessis, Farah Otaki, Shroque Zaher, Nabil Zary, Ibrahim Inuwa, Ritu Lakhtakia

**Affiliations:** 1 College of Medicine Mohammed Bin Rashid University of Medicine and Health Sciences Dubai United Arab Emirates; 2 Strategy and Institutional Excellence Mohammed Bin Rashid University of Medicine and Health Sciences Dubai Healthcare City Dubai United Arab Emirates; 3 Institute for Excellence in Health Professions Education Mohammed Bin Rashid University of Medicine and Health Sciences Dubai Healthcare City Dubai United Arab Emirates

**Keywords:** action research, change management, COVID-19, curriculum content, curriculum delivery, distance-learning, learning, medical education, pandemic, teaching

## Abstract

The COVID-19 pandemic has forced universities worldwide to immediately transition to distance-learning. Although numerous studies have investigated the effect of the COVID-19 pandemic on universities in the Middle East, none have reflected on the process through which medical education programs for health professions underwent this transition. This study aimed to elucidate the rapid transition to distance-learning of an undergraduate medical program at the College of Medicine, Mohammad Bin Rashid University of Medicine and Health Sciences (Dubai, United Arab Emirates), owing to the COVID-19 pandemic. An action research approach constituted the foundation of this collaborative effort that involved investigations, reflections, and improvements of practice, through ongoing cycles of planning, acting, observing, and reflecting. Efforts of transitioning to distance-learning were grouped into four interrelated aspects: supporting faculty members in delivering the program content, managing curriculum changes, engaging with the students to facilitate distance-learning experiences, and conducting web-based assessments. Challenges included the high perceived uncertainty, need for making ad hoc decisions, lack of experiential learning and testing of clinical skills, and blurring of work-life boundaries. Our preliminary findings show the successful generation of a strong existing digital base, future prospects for innovation, and a cohesive team that was key to agility, rapid decision-making, and program implementation.

## Introduction

The COVID-19 pandemic occurred in a globalized world. It has disrupted lives after its initial report by the World Health Organization (WHO) Country Office in the People’s Republic of China on December 31, 2019, as “pneumonia of unknown etiology detected in Wuhan City, Hubei Province of China” [1]. Initially, on January 30, 2020, the WHO declared the disease a public health emergency of international concern. On March 10, 2020, the United Nations International Children’s Emergency Fund sent an alert to protect students, and soon thereafter, on March, 11, 2020, the WHO declared COVID-19 a pandemic [2]. Countries made efforts to promote the use of personal protective equipment and impose restrictions on people’s movement to safeguard the health of their citizens. Human activity in all sectors was debilitated, and the education sector was among those most severely affected. Two major interrelated threats presented to global medical education: continuity of quality education and the resultant impact on graduating physicians’ future performance.

Toward the end of 2020, reflective studies on actions taken at educational institutions for health profession–related undergraduate and postgraduate programs have dominated the literature on medical education. Resource-rich academic environments highlighted social distancing, seclusion, and struggle with digital transformation as their largest challenges. Among resource-poor surroundings, the lack of e-learning capacity (including infrastructure, skills, learning, and development), internet affordability, connectivity, and electronic skills were the most prominent challenges [3,4].

Despite these challenges, many centers were quite innovative in overcoming deficiencies and circumventing challenges. In postgraduate and residency programs, fostering of a community of learning by using multiple educational tools enabled by proprietary platforms, including Microsoft Teams and Zoom, led the transition to distance-learning. This was a significant transition from the previous random but lesser reliable short communications within the medical resident community through social media platforms [5]. In particular, medical students on the verge of graduation were most affected, but leading institutions worldwide reoriented assessments with a web-based teaching-learning approach, complemented by open-book examinations, thus allaying students’ career-related anxiety [6].

The most prominent and impactful changes have been the initial rapid adaptation to distance-learning owing to the short lead time, and the mitigation of educational strategies that were devised and implemented during the period of complete lockdown across countries worldwide. This could be referred to as the “first wave” academic response to the first wave of the pandemic. The timeframe extended from the abrupt onset of the pandemic, blending with a sustained initial period, and lasted several months.

Although numerous studies have investigated the effect of the COVID-19 pandemic on universities in the Middle East [7], none have reflected on the process through which medical education programs for health professions transitioned [4,8]. Accordingly, the purpose of this study is to trace the abrupt educational transition of a new medical institution in the early years of its evolution, growing and delivering an undergraduate medical curriculum, on the eve of a complete nationwide lockdown in the United Arab Emirates. An in-house, cross-functional team of researchers collaborated to control for this process and document the experience in a scientific manner. This team comprised representatives of the university’s administrative workforce who handle the Quality Assurance and Institutional Effectiveness portfolio, faculty members, academic leaders, and medical education experts. In alignment with the recommendations of a scoping review of the literature on COVID-19 [9], this study elucidates a holistic multidisciplinary approach to mitigate the impacts of the COVID-19 pandemic, whose implications reach far beyond the biomedical risks, especially in medical education related to health professions. This study defines the elements of digital technology preparedness and of agile systems and identifies the initial challenges and tribulations and the subsequent triumphs of the transition to distance-learning. Finally, this study of a leap of faith in the education sector lays the foundation for a critical analysis of the challenges, gains, and lessons learned, which have allowed for consolidation and future risk-planning.

## Methods

### Context of the Study

As the most globalized country in the Middle East, the United Arab Emirates announced the first case of COVID-19 on January 29, 2020 [10]. As part of the proactive measures implemented to slow the spread of COVID-19, all educational activities in the United Arab Emirates were suspended temporarily on March 8, 2020, which was 3 days before the WHO declared COVID-19 a pandemic. Under directives of the Minister of Education, the College of Medicine (CoM) at the Mohammed Bin Rashid University of Medicine and Health Sciences (MBRU) transitioned all educational activities (including teaching, assessment, and administrative activities) completely on the internet and resumed activities in 2 weeks (as of March 22, 2020), with all employees (faculty and staff) working remotely.

The bachelor of medicine, bachelor of surgery (MBBS) program at the CoM is a 6-year undergraduate program that follows a spiral curriculum and is divided into three sequential phases: foundational basic sciences (Phase 1), preclinical (Phase 2), and clerkship (Phase 3). Phase 1 takes place over the first academic year and introduces students to basic concepts in medicine, while Phase 2 covers academic years 2 and 3 where teaching is centered around organ systems and is integrated with clinical medicine. Years 4-6 constitute Phase 3, where students undergo their clinical placements or rotations during the first 2 years and an internship in the final year.

Before the onset of the COVID-19 pandemic, the curriculum was delivered on site, in person, supplemented (where appropriate) with asynchronous assignments and activities on digital platforms. The institution has invested in several digital platforms. The first one is the “Desire-to-learn” platform, which is a learning management system (LMS) that constitutes the repository of course files, and was also actively used for forums and quizzes across all phases. A virtual microscopy–enabled website “PathXL” was actively employed for practical pathology teaching and skill-testing in Phase 2. Furthermore, the Aquifer web-based platform provided an opportunity to supplement clinical-focused problem-solving among students in Phase 3. Clinical teaching activities included simulated learning on mannikins, followed by direct patient contact in hospitals and clinics. Assessment of cognitive learning required students to be physically present at the examination center; however, it was conducted entirely on the internet, using an examination software. Objective structured practical examinations in preclinical courses were conducted on the laboratory bench and through a web-based virtual microscopy teaching and learning platform. Clinical skills were assessed in multiple formats, including case-based discussion, clinical evaluation exercise using Mini-CEX [11], and Objective Structured Clinical Examination (OSCE).

Educational activities were suspended 8 weeks into the 15-week second semester for students in preclinical years 1-3, almost at the end of midsemester in-course assessments. During this time, year 4 students were midway through the fourth of a total of 5 clinical rotations for the academic year. In the respective academic year, the enrollment numbers were as follows: 65 students in year 1, 60 in year 2, 38 in year 3, and 47 in year 4. In terms of instructors, a total of 25 clinical and nonclinical academic faculty members were teaching in the basic sciences domain, and 11 were teaching in clinical sciences domain (2 of whom taught on part-time basis). The faculty members were also coordinating and overseeing the on-site clinical rotations, while a small number of adjunct clinical faculty members, across all disciplines, were also engaged to varying degrees in the hospital setting.

The transition was characterized by a short, intense, latent period of approximately 15 days of reorganizing, regrouping, and reinforcing governance and the educational process and its delivery [12-15]. The university’s learning and teaching, research, and community engagement, through action research strategies, was structured to effectively meet the challenges of delivering its educational mission. This was achieved through problem selection, analysis, action design, implementation, and evaluation by collaborative cross-disciplinary teams of stakeholders [16]. Action research, in this context, enabled concrete and practical problem-solving and deeper reflection processes through stakeholders’ participation in research-based discourses [17-19]. Systems rapidly attempted to enable infrastructure and digital skills, which improved incrementally, as experience and troubleshooting became an integral part of the change. Thus, early intervention primed by a digitally enabled new medical institution pivoted the educational enterprise in a favorable direction. Throughout the period, leading to the conclusion of the academic year, the transition was regularly punctuated by policy guidance within the country’s health and education regulatory framework.

This situation necessitated a rapid response and concerted effort from all university spheres to ensure continuity in university operations. Empowering faculty and staff to deliver distance education while reassuring and engaging students was vital in managing the transition and successfully completing the academic year. Constant communication within and among higher and middle management, frontline employees, and academic and nonacademic organizational units was identified as key to synchronizing the educational metamorphosis.

### Action Research Approach

The classical model of action research proceeds in a series of steps that start with the general idea and involve extensive fact-checking from first-hand experience [20]. The iterative process of action research takes the form of a spiral of steps of planning, acting, and observing and reflecting [21,22]. This ongoing cycle of action research constituted the foundation of the rapid transition of the MBBS program to distance-learning. By virtue of design [20,23], the adopted action research approach was conducted by, with, and for people, rather than being directed toward people [24]. Accordingly, the university set up a COVID-19 taskforce that steered the transition and guided the operational aspects of education delivery in discussion with academic leadership.

Three months after the transition, to evaluate the experience from the perspective of the students and the faculty, the MBRU organizational unit that handles the Quality Assurance and Institutional Effectiveness portfolio (ie, the Strategy and Institutional Excellence department) assembled a data collection tool (throughout June 2021) that was contextualized to match the intricacies of the situation [15]. This tool was developed after thorough consideration of other similar tools assembled by other universities. It was first deployed at another college of the same university, during which it proved to be reliable and valid (as revealed through the Cronbach α test and principal component analysis) [13]. This tool was composed of 5 components that were measured with a 5-point Likert-type scale (1=“strongly disagree,” 2=“disagree,” 3=“neutral,” 4=“agree,” and 5=“strongly agree”). The first 4 components correspond to clarity of the explanations concerning the transition, effectiveness of the utilized information technology (IT), support received and opportunity to voice one’s opinion, and web-based material and resources. The final component assessed the perception of both groups of stakeholders of the transition experience (as a whole). In the context of our study, the tool proved to be internally consistent and externally valid [25].

## Results

### Planning Phase: Determining the Objective of the Transition and the Path and Means Toward Attaining It

The planning phase of the adopted action research approach was ultrarapid and constituted a narrow 2-week period, during which the objective of effectively transitioning to distance-learning was clarified, the path to the goal and the available means were determined, and a concrete strategy of action was developed. The strategic approach centered around ensuring the completion of the planned curriculum delivery and assessment for the academic year with reasonable modifications, upgrading digital resources, upskilling and supporting faculty, staff, and students, and ensuring safety by complying with health and education regulatory bodies. Procurement of additional digital resources and faculty and student onboarding were assigned top priority. The institution identified and invested in Microsoft Teams as the digital medium of choice for remote teaching. The acting phase involved the initiatives used to implement the action research strategy.

### Acting Phase: Transitioning of the Undergraduate Medical Program at MBRU to Distance-Learning

**Figure 1:**
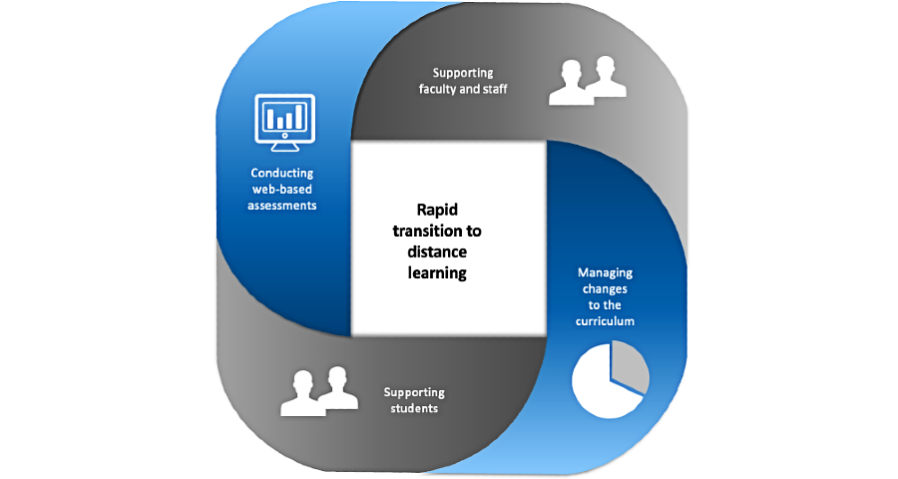
Four interrelated aspects of the transition from on-site learning to distance learning (ie, acting phase of the adopted action research approach) that needed to be juggled simultaneously.

The acting phase of the adopted action research approach was centered around 4 interrelated aspects (Figure 1).

#### Supporting the Faculty in Curriculum Delivery Through Distance-Learning

Prior to the onset of the COVID-19 pandemic, faculty development was a year-round activity targeted to identifying areas of development and conducted both by colleges and the Institute for Excellence in Health Professions Education (ieHPE). The ieHPE is the first of its kind in the Middle East, moving beyond traditional departmental and disciplinary compartmentalization to create new knowledge, enable capacity-building, and promote knowledge translation. It involves education and capacity-building, research and scholarship, and community outreach and engagement. During the transition, the CoM, with support from the ieHPE and the Smart Services and Projects (SSP) units, developed and implemented a series of initiatives to support faculty members in effectively delivering distance education. The SSP is the MBRU arm that handles all the needed IT support, serving MBRU students, faculty, academic and administrative support staff, and alumni. The SSP is composed of several units that collaborate to provide comprehensive IT services (eg, operating the LMS and all digital education and assessment tools and overseeing the university-wide evaluation system and the intranet) as well as customer service (eg, IT Help Desk and IT project management and delivery) to the MBRU community at large. There was a sharp focus on remote digital upskilling with short intense teaching and learning modules and a 24/7 Microsoft Teams–anchored community-of-learners helpline. The initiatives that were meant to support the faculty throughout the transition consisted of the following activities.

##### Raising Faculty Awareness of Available Resources to Support Distance-Learning

An immediate needs assessment survey was assembled to gauge faculty members’ familiarity with web-based teaching, expectations, requirements, and level of assistance required. The ieHPE subsequently organized sessions that explained the paradigm of web-based education and contrasted it to traditional classroom-teaching. The different modalities to be used, such as synchronous web-based delivery of didactic sessions and prerecording didactic sessions by creating screencasts, and podcasts were also advocated. The SSP shared with faculty members the available resources that could support such pedagogical modification.

##### Faculty Learning and Development

The staff at the ieHPE organized for and delivered hands-on workshops and “drop-in” sessions for faculty members to consult for the optimal learning and teaching configurations depending on the nature of the teaching session, as well as modifying teaching approaches to suit distance-learning. Specific hands-on training was provided using Microsoft Teams and Live Lecture Capture. Any hardware updates and modifications needed by the faculty members were also provided by the SSP team. Additional support and training were provided to faculty members to maximize engagement with learners on the internet, including creating live asynchronous classroom and discussion forums.

##### Supporting the Mental Health of the Faculty and Staff

Numerous measures were implemented to ensure that all MBRU employees (faculty and staff) are supported in terms of their mental health. Initially, all employees had access to the university counselor and were encouraged to reach out to the counselor’s office to schedule an appointment when required. In addition, the counselor offered weekly web-based group relaxation sessions, which were open to all employees. Furthermore, stand-up meetings continued as usual to maintain cohesion and interaction among colleagues (which also indirectly played a protective role in the employees’ mental health). Finally, committee chairpersons and Phase Directors supervised all employees and provided enhanced support throughout the experience.

#### Managing Changes to the Curriculum

Under the auspices of the Office of the Dean of the CoM and with the assistance of the Curriculum Committee, the academic calendar was immediately adjusted, and schedules were revised. This included preponing the spring break by 2 weeks to create space for preparation. Together with the accompanying modifications of roles and responsibilities, these calendar changes were instantly communicated to instructors. Implementation was regularly monitored by Phase Directors and course coordinators. Simultaneously, weekly meetings were scheduled with the dean of the CoM to discuss progress at the college level and with the respective academic committees’ chairpersons to share updates and directives from the Ministries of Public Health and of Higher Education. As part of the implementation phase, a gap analysis was performed to ascertain the impact of reverting to web-based learning on the curriculum outcomes of the respective phases. This included measuring course learning objectives achieved and comparing them to those of the respective courses in accordance with the programs and study guides. All objectives set for Phase 1 (which aims at introducing students to basic concepts in medicine) were met, while 1 course (ie, “Foundations of Clinical Medicine-IV”) in Phase 2 was significantly affected since it is designed to foster learning of experiential clinical skills at the university’s simulation center. The impact was most significant on the Phase 3 curriculum since students could not complete the last 2 clinical rotations; however, all didactic teaching was carried out on the internet. Concerted efforts were made to compensate for the lost clinical experience through case study–based Aquifer sessions and web-based case-based discussions. Longitudinal COVID-19 rounds, led by clinicians in the hospital, were also conducted every week. This initiative was an innovative educational approach, where a group of students (on a rotating basis) would address a particular aspect of COVID-19 and its updates (eg, socioeconomic factors or medication) and collate them as an all-encapsulating infographic. During the session, the assigned team facilitated discussions were centered around the infographic. Considering the gaps, it was decided to introduce a 3-week “enhanced induction” at the beginning of the following academic year, which was intended to address the identified deficiencies for all students in Phase 3.

#### Supporting Students During Web-Based Learning

##### Communication With the Students

The Student Services and Registration (SSR) organizational unit was instrumental in communicating and updating students throughout this period. This included highlighting changes to the schedule, sharing of ministerial directives, changes in examination modalities, and the implementation of the “pass” or “fail” option. Course coordinators were tasked with sharing course-specific changes and weekly planning schedules.

##### Students’ Connectivity and Readiness

The SSR surveyed students to determine their ability to fully participate in web-based activities by requesting the specifications of the devices that the students would use to connect on the internet, as well as the stability and bandwidth of their internet connection. Access to Microsoft Teams and training was also provided during subsequent usage. Continuous IT support was also made available to all students.

##### Students’ Connectedness and Engagement

Student engagement prior to the pandemic was monitored through an established in-class attendance record, academic advisor meetings with digital records and follow-up, and meetings with Phase Directors and assessment chairs.

During the transition, all learning material was shared on the LMS, well in advance of the web-based sessions. Simultaneously, students received weekly updates from course coordinators with regard to the course schedules and presentation modes. Individual instructors posted expectations as well as formative assessments for sessions on the LMS.

Student engagement was further monitored through real-time logging on to the synchronous web-based sessions as well as extracting data of their engagement with learning material on the LMS. Course reports were compiled weekly, and those students who did not engage adequately were directly contacted and encouraged to improve their participation. The SSR also followed up with these students to determine any underlying reasons for their insufficient engagement (eg, connectivity issues or personal hurdles). Appropriate action was then taken. Academic advisors were also vigilant in engaging their advisees for early identification of challenges and providing prompt support to mitigate adverse outcomes.

##### Students’ Health, Well-being, and Mental Health Support

Prior to the pandemic, several agencies supported the students’ academic and nonacademic needs including but not limited to academic advisors, Office of the Assistant Dean of Student Happiness and Wellbeing, the SSR, and the student counselor. Each of them had independent and interdependent functions. On-campus life was steered by leadership of the student council and a host of extracurricular activities through student clubs.

Owing to the anticipated burden of deviating from the known traditional on-campus to complete off-campus remote teaching and learning on the internet, and the uncertainty of the psychosocial effects of the pandemic on faculty and students, it was also important to look after the health and well-being of all the community members, especially the students. The SSR, together with the students’ council, scheduled various web-based extracurricular activities to support and maintain a sense of community among the student body. Furthermore, the student counselor developed a series of relaxation sessions and sessions aimed at equipping faculty and students with internal resources and coping mechanisms to deal with anxiety and stress.

A peer-mentoring program was also implemented. Since students in Phase 3 could not return to their clinical placements, part of their schedule was freed up, and volunteers were recruited to tutor students, particularly those in Phase 1. This served as a support system to the freshman students and provided senior students with a sense of purpose.

Toward the end of the academic year, it was decided to provide students with an extended study break before the final examination to provide them sufficient time to consolidate learning material and prepare for examinations.

#### Conducting Web-Based Assessments

The university had initially invested in a proprietary web-based examination platform (ie, ExamSoft) at the launch of the MBBS program, which was used for student assessment on campus before the onset of the pandemic. On the same platform, end-semester and end-year examinations and other forms of student assessment were delivered remotely during May-July 2020. The transition to web-based examination was therefore smooth as a remote proctoring tool was added to the existing digital platform to ensure academic integrity. This was deduced from the large proportion of class learning objectives met; student performance (assessment and progression), which did not differ from previous iterations; and student and instructors’ satisfaction with the rapid transition to distance-learning in the end-of-course surveys [15]. However, the main challenge was ensuring proper identification of examination takers and avoiding unauthorized student access to material during examinations. Accordingly, an additional capability of remote proctoring was added to the examination platform to ensure the integrity of the assessment conducted remotely. Moreover, modified electronic versions of the OSCE generated in-house and video case-based evaluations were effective as the best fit for purpose in a remote setting.

### Observing and Reflecting Phase: Challenges and Triumphs

Disruption of education was not an isolated phenomenon during the pandemic, and its acuteness was most palpable at the onset of the forced transition. Despite extensive efforts made at all levels, uncertainties created by educational directives that were in turn dictated by rising infections caused varying communication delays across the board. Overall, effects on mitigating the fear of infection and coping with isolation had to be balanced with the need for continuity in education. As expected, despite close monitoring and support, vulnerable individuals and borderline performers were most impacted more through academic stress than measurable on performance.

Nonetheless, several short- and long-term gains have been made. The digital efficiency enabled curriculum delivery and administrative meetings to achieve heightened focus, brevity, and timeliness. Recordings afforded flexibility, archiving, and efficient use of time, and live sessions provided impetus for innovative web-based activities. As 1 year of living with the COVID-19 pandemic has been completed, interesting and beneficial changes have persisted. In the context of our institution, these abiding changes include a digital revolution, personalized certifications in digital teaching, hybrid teaching, and adaptation of the lessons learnt from the electronic version of the OSCE to undertake electronic multiple mini-interviews for new admissions to programs. In terms of the stakeholders’ perception of the experience, both groups appeared quite satisfied. The total average of satisfaction among stakeholders was 76.4% [25].

## Discussion

### Principal Findings

The COVID-19 pandemic created a window of opportunity for action research in medical education. Similar to any other action research study [18,19], the outcome of transitioning the MBBS program to distance-learning at the CoM was not defined a priori and resulted from the involved stakeholders’ capacities, interests, and actions. It was immediately apparent that the stakeholders and their work would metamorphose, but what form it would take could not have been predicted. MBRU values of respect, integrity, connectivity, giving, and excellence [26] enabled the entire process by focusing on its core and acted as the stakeholders’ compass throughout the journey. Leveraging the internal resources, including but not limited to the existing IT infrastructure and support team (ie, the SSP), and the internal expertise in medical education related to health professions (ie, ieHPE) were also fundamental to the transition. As such, the changing public needs owing to the COVID-19 pandemic were addressed by deploying an action research approach to restructure the university and its relationships and fostering the key positive elements of MBRU.

The United Nations Educational, Scientific and Cultural Organization defines an “educational emergency” as a crisis that is created by conflicts or disasters that have destabilized, disorganized, or destroyed the education system and requires an integrated process of crisis and postcrisis support, recognizing the importance of ensuring education continuity after disasters, and taking the lead in promoting education as part of an emergency response and for long-term recovery [27]. The impact of the COVID-19 pandemic on education was an unexpected bio-disaster. Adaptation to the changed circumstances and mitigation of its impact required known and yet unknown resources to devise solutions. Through the process adapted in this study, it was evident that 4 interrelated aspects of the transition needed to be closely followed up: managing the supporting faculty members in delivering the curriculum, managing curriculum changes, engaging with the students to facilitate the distance-learning experience, and conducting web-based assessments. This study bridges a gap in the literature by elucidating a process through which a medical university in the Middle East leveraged its internal resources to abruptly transition an MBBS program to distance-learning.

The first educational responder was China, where the pandemic originated, which did not benefit from reviewing coping strategies with to this specific threat. In contrast, other countries had a 3-month lag period before being affected by COVID-19. In an insightful case study from Peking University, an educationist reflected that 5 high-impact principles of web-based education served them well, including “(1) high relevance between online instructional design and student learning, (2) effective delivery on online instructional information, (3) adequate support provided by faculty and teaching assistants to students, (4) high-quality participation to improve the breadth and depth of student's learning, and (5) contingency plan to deal with unexpected incidents of online education platforms” [28]. At MBRU, the navigation of the curriculum retained the intended design and delivery as those of prepandemic electronic platforms for teaching and assessments. This required escalating efforts toward stabilizing capacity through rapid, expedited faculty development on additional electronic tools to facilitate continuity in teaching and keep it engaging. Investment in the identified Microsoft Teams platform and student orientation provided sustainability.

Part of the previous reluctance across the medical professions toward remote learning is the perception of the inability to effectively deliver practical learning. Nonetheless, institutions surmounted such obstacles, where, for example, teaching of anatomy at universities in Australia and New Zealand balanced the loss of “hands-on” experience and pedagogy with “six critical elements” that include “community care, clear communications, clarified expectations, constructive alignment, a community of practice, ability to compromise, and adapt and continuity planning” [29]. The use of a blended pedagogical framework through a social media application–integrated “interactome” strategy proved useful during the pandemic when teaching anatomy at MBRU [12]. Interestingly, the usage of MUELE, the official e-learning platform used at Makerere University, was much lower at their College of Medicine compared to other colleges at the same university [3]. In the transition reported in this study, there was minimum interruption in the first 3 years in learning, teaching, and assessments. There was only 1 cohort in the first clerkship year, and the challenge to replace clinical on-site clinical rotations with virtual, real-time interactive sessions was a compromise at best.

Virtual learning during the COVID-19 pandemic helped reimagine and blend the well-established practices of telehealth, which had previously been limited to provide health access to remote areas, by rendering it the central focus in educational processes [30]. Virtual learning drew students’ attention to the rapidly advancing innovations in delivering home health care and the expanding inventory of handheld devices and apps that help monitor chronic ailments.

Student support was completely redefined during the acute transition to coping with isolation and learning simultaneously. High levels of anxiety and stress and the resurgence of pre-existing mental disorders identified through structured interviews were expected [31]. Addressing them through counseling and psychoeducational interventions was necessary. In our short journey, this was not left to chance, with active interaction maintained with students and at multiple levels from university leadership, academics, advisers, and counselor services. All educational functionaries also searched for new skills to deliver their respective roles in working from home with unexpected distractions from people and competition for space.

Community engagement is a vital activity of universities and students’ engagement is critical. This engagement becomes even more critical for medical students when a health disaster strikes. It becomes supplementary to curricular learning, as pandemics constitute live exposure to learning emergency medicine and public health responses [32]. An interesting case study of higher education regarding the public health response to disruption during the Christchurch earthquake of 2010 provides interesting insights in to the dynamic way service-learning made curricula responsive and engaging, turning an educational disruption into a pedagogical opportunity [33].

### Limitations

Through an action research approach, this study provides thorough reflections on a particular experience that is relevant to stakeholders of other health profession–related educational programs. By virtue of this study’s design, the generalizability of its findings is limited to institutions that are characteristically and contextually analogous to MBRU. Moreover, since the focus of this study was on the inductive process adapted by the institution to effectively respond to a crisis, it was purely descriptive. Follow-up studies are required to focus on a single institution to capture the perceptions of several stakeholders and to strive to systematically integrate quantitative and qualitative data through a mixed-methods analysis.

### Conclusions

This university-wide action research approach highlights the experience of a first responder in an educational crisis with a recently established undergraduate medical program of a young university at the outset of the COVID-19 outbreak and nationwide lockdown. Seminal triumphs of this study included building on a strong existing digital base, prospects for innovation, and a modest and cohesive team that was key to agility, rapid decision-making, and implementation. Challenges included the uncertainty of endpoints, rapid decision-making, clinical skill–learning and –testing, and blurring of work-life boundaries. This educational “leap of faith” was not based on flamboyance; instead, it relied on the strength of its purpose, a sound digital infrastructure, and focused reorientation and delivery of the curriculum. Experiences of newly devised innovations and adaptations toward multiple formats of remote assessments will help integrate the “new normal” with the “old normal” academic journey narrative. A year on, digital upscaling and upskilling and hybrid educational experiences have persisted.

## Abbreviations

CoM: College of Medicine
ieHPE: Institute for Excellence in Health Professions Education
IT: information technology
LMS: learning management system
MBBS: bachelor of medicine, bachelor of surgery
MBRU: Mohammed Bin Rashid University of Medicine and Health Sciences
OSCE: Objective Structured Clinical Examination
SSP: Smart Services and Projects
SSR: Student Services and Registration
WHO: World Health Organization
